# Polycystin-2 (TRPP2) regulates primary cilium length in LLC-PK1 renal epithelial cells

**DOI:** 10.3389/fphys.2022.995473

**Published:** 2022-10-04

**Authors:** Noelia Scarinci, Paula L. Perez, Horacio F. Cantiello, María del Rocío Cantero

**Affiliations:** Laboratorio de Canales Iónicos, IMSaTeD, Instituto Multidisciplinario de Salud, Tecnología y Desarrollo (CONICET-UNSE), Santiago del Estero, Argentina

**Keywords:** primary cilia, polycystin-2, ADPKD, calcium, lithium

## Abstract

Polycystin-2 (PC2, TRPP2) is a Ca^2+^ permeable nonselective cation channel whose dysfunction generates autosomal dominant polycystic kidney disease (ADPKD). PC2 is present in different cell locations, including the primary cilium of renal epithelial cells. However, little is known as to whether PC2 contributes to the primary cilium structure. Here, we explored the effect(s) of external Ca^2+^, PC2 channel blockers, and *PKD2* gene silencing on the length of primary cilia in wild-type LLC-PK1 renal epithelial cells. Confluent cell monolayers were fixed and immuno-labeled with an anti-acetylated α-tubulin antibody to identify primary cilia and measure their length. Although primary cilia length measurements did not follow a Normal distribution, the data were normalized by Box-Cox transformation rendering statistical differences under all experimental conditions. Cells exposed to high external Ca^2+^ (6.2 mM) decreased a 13.5% (*p* < 0.001) primary cilia length as compared to controls (1.2 mM Ca^2+^). In contrast, the PC2 inhibitors amiloride (200 μM) and LiCl (10 mM), both increased primary ciliary length by 33.2% (*p* < 0.001), and 17.4% (*p* < 0.001), respectively. *PKD2* gene silencing by siRNA elicited a statistically significant, 10.3% (*p* < 0.001) increase in primary cilia length compared to their respective scrambled RNA transfected cells. The data indicate that conditions that regulate PC2 function or gene expression modify the length of primary cilia in renal epithelial cells. Blocking of PC2 mitigates the effects of elevated external Ca^2+^ concentration on primary cilia length. Proper regulation of PC2 function in the primary cilium may be essential in the onset of mechanisms that trigger cyst formation in ADPKD.

## 1 Introduction

The primary cilium is a solitary, non-motile, sensory organelle projecting from the apical surface of renal epithelial cells. Primary cilia transduce different signals from extracellular stimuli regulating cell proliferation, differentiation, transcription, migration, polarity, and survival (Lee and Gleeson, 2010). The organelle expresses many receptors to recognize specific hormones such as somatostatin, growth factors, or morphogens, including Sonic hedgehog (*Shh*) and *Wnt*, which play essential roles in the embryonic phase (Fliegauf et al., 2007), and abundant cation-selective channel activity, including PC2, TRPC1, and ENaC (Raychowdhury et al., 2005). A direct relationship has been found between the morphology of the primary cilium and the pathogenesis of several diseases known as ciliopathies, caused by defects in the formation or function of cilia (Wheatley, 1995). Ciliopathies render a group of clinical syndromes sharing common phenotypic features. There are more than a hundred known ciliopathies to date, including syndromes such as Bardet-Biedl (BBS), Joubert (JBTS), Meckel-Gruber (MKS), Alström, Senior-Löken, and Oro-facial-digital type 1 (OFD1), and diseases such as retinitis pigmentosa, ADPKD, autosomal recessive polycystic kidney disease (ARPKD), nephronophthisis (NPHP), abnormalities in axial embryonic asymmetry, obesity, and cancer (Badano et al., 2006; Fliegauf et al., 2007; Valente et al., 2014), with a collective frequency of 1:1000 (Davis and Katsanis, 2012). The vast majority of mutated proteins linked to renal cystic diseases localize to the primary cilium and associated structures, including the basal body, the centrosomes, and the ciliary transition zone (Hildebrandt and Otto, 2005). However, little information is available on the role morphological changes in the primary cilium play in the highly variable renal phenotypic spectrum seen in different diseases, which range from dysplasia to degeneration or fibrosis to cysts, and remain to be defined patients with cystic disease (Siroky and Guay-Woodford, 2006; Ong, 2013).

Mutations in either of two genes, *PKD1* or *PKD2,* cause ADPKD, characterized by developing epithelial-lined cysts in various organs, including the kidney and the liver (Hopp et al., 2012). The protein products of *PKD1* and *PKD2* are transmembrane proteins called polycystin-1 (TRPP1, PC1) and polycystin-2 (TRPP2, PC2), respectively, whose localization to primary cilia was an essential clue in directly linking primary cilia to renal cysts (Yoder et al., 2002). ADPKD, in particular, is thought to develop by dysfunction of a PC1/PC2 functional receptor/ion channel complex present in various cell locations, including the primary cilium (Ong, 2013; Ma et al., 2017). Changes in the length of the primary cilium occur in tissues and cells grown under various physiological and pathological conditions (Pazour et al., 2000; Smith et al., 2006; Verghese et al., 2008; Ou et al., 2009). The length of renal primary cilia increases in ischemic mouse kidneys and in human kidney transplants that suffer acute tubular necrosis (Verghese et al., 2008; Verghese et al., 2009). Renal tubular primary cilia also lengthen in several hypomorphic mouse mutants (Smith et al., 2006; Bergmann et al., 2008; Hopp et al., 2012), and abnormally long primary cilia are associated with juvenile cystic kidney disease (Smith et al., 2006). Conversely, in the mouse model with partial loss of the ciliary protein Polaris, the animals died of ARPKD, and the kidneys showed abnormally short primary cilia (Pazour et al., 2000). There is no consensus as to the functional consequences of cilia elongation in comparison to cilia shortening in ADPKD (Ong, 2013). *Pkd1* and *Pkd2* heterozygous mice are more sensitive to acute ischaemia-reperfusion renal injury than their wild-type littermates (Bastos et al., 2009; Prasad et al., 2009). Harris’s group reported that cystic collecting duct epithelia in the *wpk* and MKS3 patients displayed longer (Tammachote et al., 2009), or slightly shorter cilia in human *PKD1* cystic epithelia (Nauli et al., 2003; Xu et al., 2007). Cilia length and function appear normal in the *inv* model (NPHP2) (Shiba et al., 2005) and in *Pkd1* ^−/−^ cells. The *PKD* animal models also show different responses, including shorter cilia in mutant Pkhd1 biliary epithelia (Masyuk et al., 2003; Woollard et al., 2007) and the Tg737-*orpk* mouse model (Pazour et al., 2000). In constrast, longer cilia were observed in the *jck* (Smith et al., 2006), *Nphp3−/pcy* (Bergmann et al., 2008) and Mks3 mouse models (Cook et al., 2009) while cilia more variable in length were reported in the *cpk* model (Muchatuta et al., 2009). Ciliary structure, function, and stability play essential roles in normal kidney development, and it appears that the *PKD* associated proteins may have different regulatory roles on the length of primary cilia. The mechanisms of cyst formation in the different genetic diseases implicate cilia dysfunction, including abnormalities in cilia structure, composition, and signaling (Watnick and Germino, 2003; Hildebrandt and Zhou, 2007). In particular, the polycystin receptor-channel complex may function poorly on cilia of inappropriate length that result in cyst development.

The mechanosensor hypothesis of primary cilia function suggests that changes in environmental cues such as fluid flow may be recognized and transduced by primary cilia to elicit cell signaling (Praetorius and Spring 2001; Nauli et al., 2003). These signals trigger the response of the PC1/PC2 receptor-channel complex, where a functional PC2 channel in the primary cilium of renal epithelial cells may elicit the initial Ca^2+^ response (Raychowdhury et al., 2005). However, it remains to be determined how this system works. The mechanosensory properties of primary cilia in kidney cells has been questioned along with PC1’s function as a flow sensor (Nauli et al., 2003) because no ciliary Ca^2+^ influx was observed consistent with mechanosensitive channel activation (Delling et al., 2016). It has been suggested that if the mechanosensory ability of primary cilia does exist, it could induce mediators and not initiate cell Ca^2+^ signaling (Ma et al., 2017). It is presently uncertain whether the exclusive “cilia hypothesis” can explain the highly variable renal phenotypic spectrum seen in different diseases (Loftus and Ong, 2013).

PC2 is a Ca^2+^-permeable nonselective cation channel of the TRP channel superfamily (González-Perrett et al., 2001) implicated in the Ca^2+^ entry step of different epithelial tissues and organs (González-Perrett et al., 2001; Luo et al., 2003; Tsiokas et al., 2009; Narayanan et al., 2013; Zhao et al., 2015). PC2 contribution to cell signaling and Ca^2+^ homeostasis has also been reported in separate cellular compartments, including the primary cilium (Koulen et al., 2002; Tsiokas et al., 2009; Zhou, 2009). As a TRP channel, PC2 is a functional homo-tetramer that does not require a complex with PC1 to function, but can hetero-multimerize with other partners, including PC1, which interacts *via* the coil-coil domains of their respective carboxy terminal. More recently it has been shown that this hetero-oligomerization may include at least one monomer of PC1 (Wang et al., 2019). Walker et al. (2019) demonstrated that altered ciliary localization of PC2 is sufficient to cause cystogenesis, such that a possible docking of PC2 at the base of the cilium may be essential for its ciliary localization and control of ciliary-dependent cyst-formation. In the context of the ADPKD ciliopathy, in particular, little is known as to the possible link(s) between PC2 channel function and the morphology of the primary cilium.

Here, we explored different conditions that modulate either PC2 channel function or expression on the length of primary cilia in LLC-PK1 renal epithelial cells. Under physiological external Ca^2+^ conditions (1.2 mM), primary cilium length is maintained by PC2-mediated Ca^2+^ entry. In a high external Ca^2+^ concentration (6.2 mM), the length of the primary cilium was reduced, which was consistent with an increased intraciliary Ca^2+^ and the depolymerization of axonemal microtubules. Either PC2 inhibition by Li^+^ (Cantero and Cantiello, 2011), amiloride (González-Perrett et al., 2001) or reduction of its expression (siRNA) (Dai et al., 2017), would instead render longer primary cilia, in agreement with the hypothesis that a reduced Ca^2+^ entry to the organelle decreases the rate of MT depolymerization. The results indicated that a functional PC2 is a critical element in regulating the length of the primary cilium in LLC-PK1 renal epithelial cells, which may help explain the initial events in cyst formation and the onset of ADPKD.

## 2 Materials and methods

### 2.1 Cell culture and immunochemistry

**FIGURE 1:**
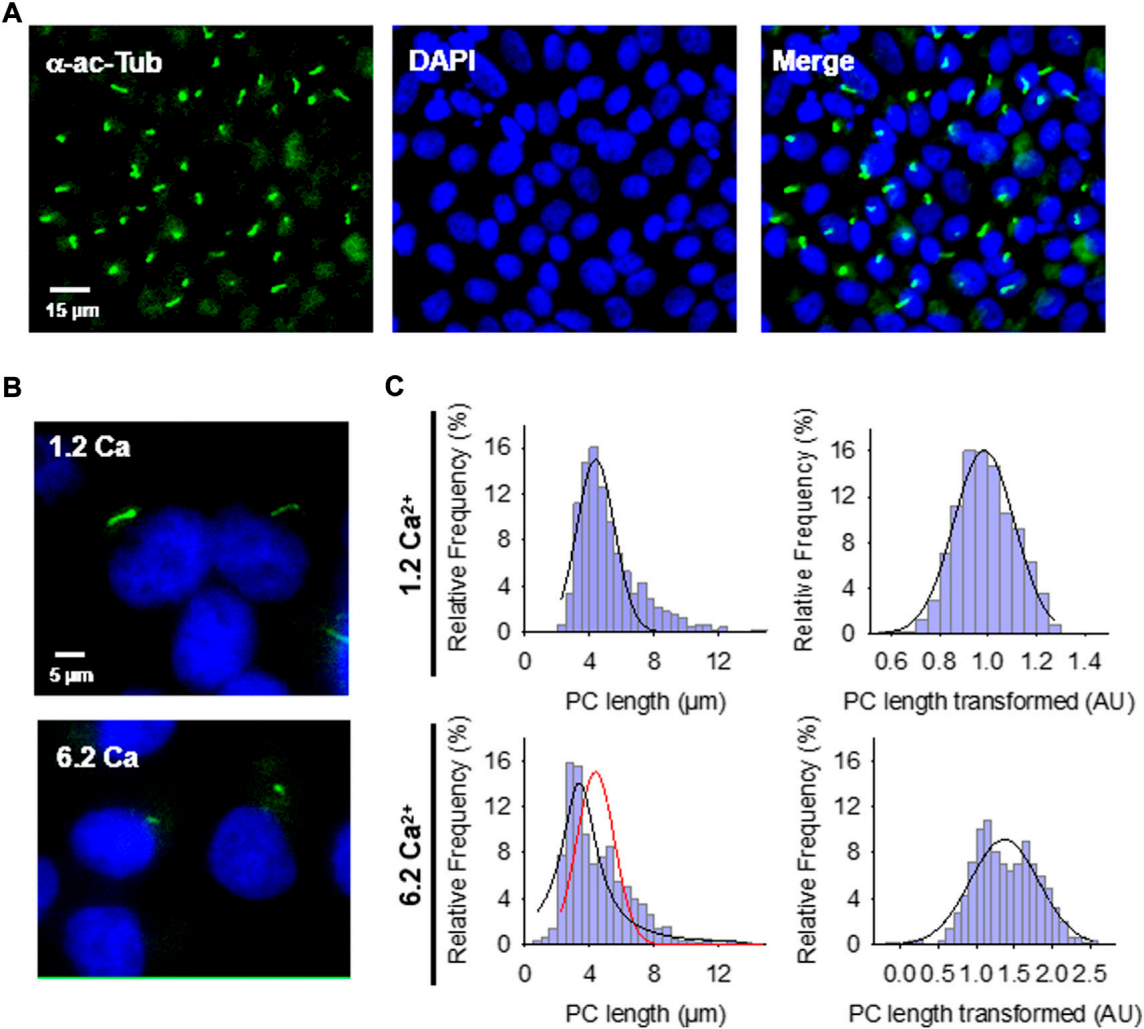
Effect of extracellular Ca^2+^ on the primary cilium (PC) length of wild-type LLC-PK1 cells. **(A)** Confluent monolayers of wild-type LLC-PK1 cells were labeled with an anti-acetylated α-tubulin antibody (Green, Left Panel), and DAPI (Blue, Middle Panel). The merged image is shown on the Right. **(B)** Acetylated-α-tubulin immunolabeling of confluent of LLC-PK1 cell monolayers was used to observe the primary cilium at normal (1.2 Ca, Top panel) and high (6.2 Ca) external Ca^2+^ concentrations. Merged images also show cell nuclei by DAPI labeling (Blue). **(C)** Histograms of ciliary length measurements in standard (1.2 mM, Top) and high (6.2 mM, Bottom) Ca^2+^ concentration are shown before (Left) and after (Right), Box-Cox transformation. A not Normal left-skewed distribution of data is observed before the transformation. Please note that Box-Cox transformed histograms require re-transforming the values to render primary cilia length in µm (see Materials & Methods). Red line indicate control distribution.

Wild-type LLC-PK1 renal cells (ATCC) were cultured as previously described (Raychowdhury et al., 2005) in Dulbecco’s modified Eagle’s medium (DMEM) supplemented with 3% fetal bovine serum (FBS) without antibiotics. Cells were seeded onto glass coverslips and grown at 37°C in a humidified atmosphere with 5% CO_2_ to reach full confluence in two to 3 weeks in culture. Confluent cells were used for immunocytochemical studies (Figure 1A, Supplemental Material).

### 2.2 Immunocytochemistry

Confluent cell monolayers exposed overnight to the various experimental conditions were rinsed twice with phosphate-buffered saline (PBS) and fixed for 10 min in a freshly prepared solution of paraformaldehyde (4%) and sucrose (2%). Cells were then washed three times with PBS, blocked for 30 min with BSA (1%) in PBS, and incubated for 60 min with an anti-acetylated-α-tubulin antibody (Santa Cruz Biotechnology) to identify primary cilia (Raychowdhury et al., 2005). Anti-mouse IgG FITC-coupled (Invitrogen) was used as secondary antibody. Cells were counter-stained with DAPI to locate cell nuclei and mounted with Vectashield mounting medium (Vector Laboratories, Burlingame, CA). Cells were viewed under an Olympus IX71 inverted microscope connected to a digital CCD camera C4742-80-12AG (Hamamatsu Photonics KK, Bridgewater, NJ). Images were collected and analyzed with the IPLab Spectrum acquisition and analysis software (Scanalytics, Viena, VA), running on a Dell-NEC personal computer.

### 2.3 Reagents

Unless otherwise stated, chemical reagents, including CaCl_2_, LiCl, amiloride, and EGTA, were obtained from Sigma-Aldrich (St. Louis, MO, United States) and diluted at their final concentrations indicated.

### 2.4 Free Ca^2+^ calculations and Ca^2+^ chelation

The nominal free-Ca^2+^ solution was prepared as follows: the Ca^2+^ chelating agent EGTA (ethylene-bis(oxyethylenenitrilo) tetraacetic acid, 100 mM) was dissolved in NaOH and titrated with HCl to reach pH ∼7.0, and used at a 1 mM final concentration. The final Ca^2+^ concentration was calculated by:

$$\left[Ca^{2+}\right]=\frac{K_{Q}\times \left[CaQ\right]}{\left[Q\right]}$$ (1)

where *K_Q_* is the dissociation constant of the Ca^2+^-chelator complex, [*Q*] is the concentration of the free chelating agent, and [*CaQ*] is the concentration of Ca^2+^ bound to Q. The nominal Ca^2+^-free solution was estimated to have a Ca^2+^ concentration of 0.32 nM. Whenever indicated, CaCl_2_ was added from a 500 mM stock solution to reach either 1.2 mM (normal) or 6.2 mM (high) free Ca^2+^. The solutions were labeled as 0 Ca, 1.2 Ca, and 6.2 Ca, respectively.

### 2.5 *PKD2* gene silencing

As recently reported, silencing of *PKD2* gene expression in cultured LLC-PK1 cells was conducted using the small interfering RNA technique (Dai et al., 2017). Briefly, two 21-nt PKD2-specific synthetic siRNAs, one of which was a fluorescent (fluorescein) probe, were synthesized by Invitrogen (Buenos Aires, Argentina), as well as a 19-nt irrelevant sequence as scrambled control (Ir-siRNA). As reported initially, all constructs bore dTdT overhangs at the 3′ end (Wang et al., 2011). The siRNAs sense sequences were as follows; (siPKD2) GCU​CCA​GUG​UGU​ACU​ACU​ACA, starting at 906 in exon 3 of the porcine *PKD2* gene, and (Ir-siRNA) UUC​UCC​GAA​CGU​GUC​ACG​U, as scrambled control. siRNA transfection was conducted as follows (Dai et al., 2017): Cell cultures were trypsinized and placed at 70% confluence in 35-mm cell culture dishes containing DMEM supplemented with 3% FBS at 37°C in a 5% CO_2_ atmosphere. The following day, transfection was performed with Lipofectamine 2000 (Invitrogen). Tubes were added either scrambled (Irss, 10 µl) or antisense (*siPKD2*, 10 µl) RNA with Optimum medium (100 μl) in the absence or presence of Lipofectamine (2 μl). Briefly, the tubes were incubated for 5 min at room temperature and then mixed with either Irss or *siPKD2* for another 20 min (200 μl total volume). Incubation was conducted by medium change with a mixture of fresh medium (800 μl, DMEM plus 3% FBS) and 200 μl of the transfection mixture. The total transfection time was three overnights (72 h). Silencing efficiency was confirmed by the Western blot technique, 42 ± 7.91%, *n* = 4 reduction (Figure 3B), as originally reported by Wang et al. (2011), and previously reproduced in our laboratory (Dai et al., 2017). It is important to note that in that study, transfection efficiency was assessed in material from semiconfluent cell cultures, while single cell detection for patch clamping experiments was assessed by fluorescent siRNA uptake, to identify positive cells that had taken up the probe and likely silenced the gene product in a cell-by-cell basis. In those experiments silencing was 100% efficient (Dai et al., 2017). The siRNA-treated LLC-PK1 cells showed a 59% reduction in the whole cell currents, as compared to their own controls, Irss-treated cells, which was in close agreement with the inhibition observed by intracellular dialysis with active anti-PC2 antibody, but somewhat lower than that under Ca-free conditions, suggesting that even in the absence of external Ca^2;^, there is a background PC2 current (Dai et al., 2017). The specific *PKD2* gene expression knock-down was confirmed by real-time quantitative PCR, which was normalized to the β2MG housekeeping gene, with an average 47.9% decrease in *PKD2* mRNA. The silencing efficiency was confirmed by Western blotting with a reduction of 39% to the value obtained by qPCR. These results were similar to those reported by Wang et al. (2011).

### 2.6 Measurement of primary cilia length

The length of extended primary cilia on the fixed confluent monolayer was manually measured from 2D images with the image analysis program ImageJ (NIH software), as described elsewhere (Miyoshi et al., 2009; Ou et al., 2009; Besschetnova et al., 2010; Sipos et al., 2018). Briefly, FITC-labeled fluorescent primary cilia were traced with the “Freehand Line” tool of the software to obtain its length in pixels (Figure 1A, Supplemental Material). The results were then converted into µm by calibration with a Neubauer chamber (Hausser Scientific, Horsham, PA, United States). Although this technique risks a selection bias, it is considered reliable, allowing the measurement of many irregular primary cilia and facilitating their subsequent statistical analyses (see Supplemental Material).

### 2.7 Statistical analyses

**FIGURE 2:**
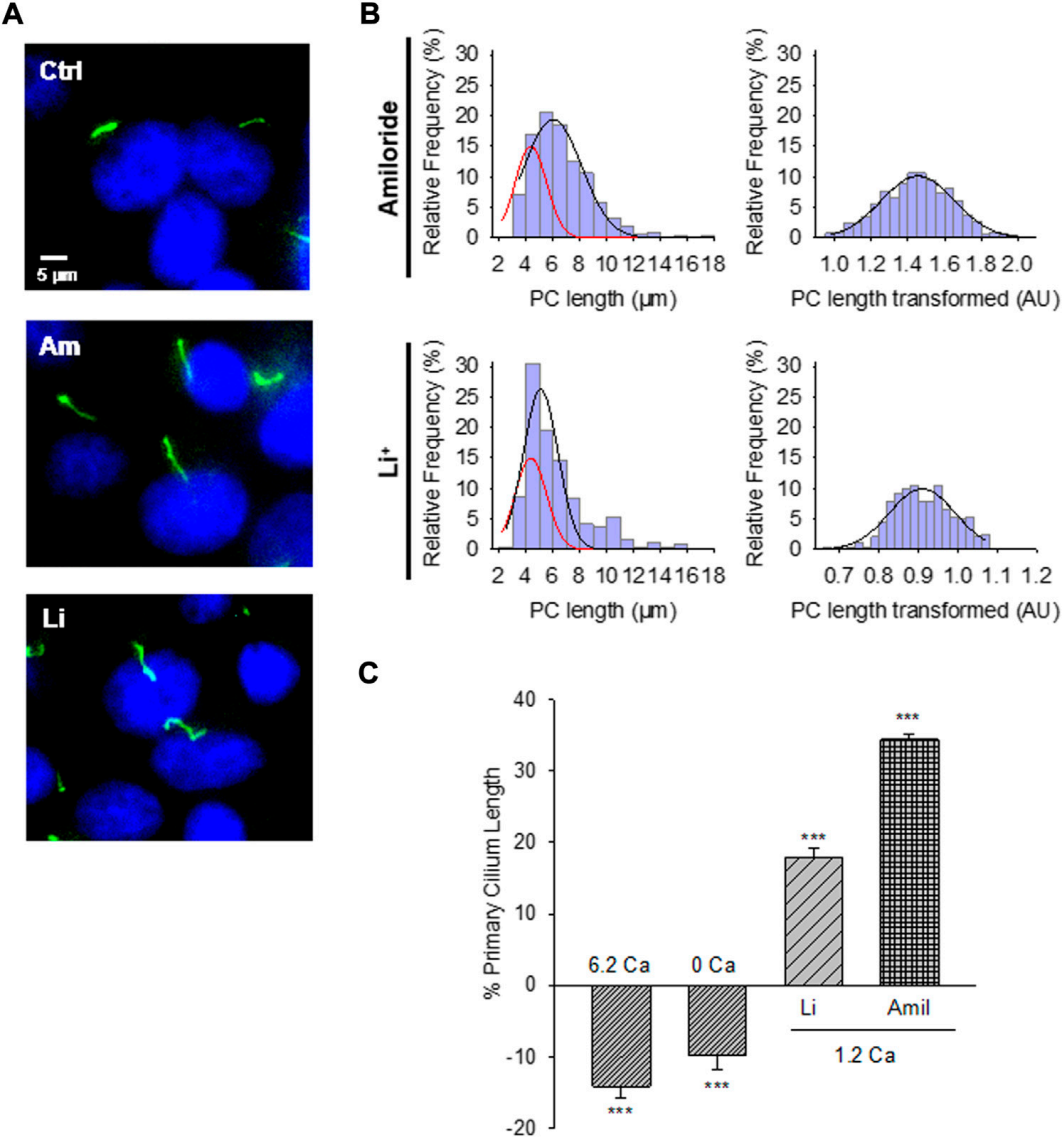
Effect of PC2 channel inhibitors on the primary cilium length of wild-type LLC-PK1 cells. **(A)** Acetylated-α-tubulin antibody immunolabeling of LLC-PK1 cells (FITC, Green) to observe the length of primary cilia under control (Ctrl, Top Panel) condition and the presence of either amiloride (200 μM, Middle Panel), or LiCl (10 mM, Bottom Panel). Cells were incubated in normal 1.2 mM Ca^2+^. Images were obtained with ×60 objective. **(B)** Red lines indicate control distribution. Histograms of ciliary length measurements in normal Ca^2+^ and the presence of either amiloride (Top) or LiCl (Bottom) are shown before (Left) and after (Right), Box-Cox transformation. A not Normal left-skewed distribution of data is observed before the transformation. **(C)** Bar graphs of percentage increase (positive bars) or decrease (negative bars) in the length of the primary cilium under various conditions, including Free- and high (6.2 mM) Ca^2+^ external conditions, as well as incubations with LiCl (10 mM) and amiloride (200 μM) respect to the 1.2 Ca condition. Li^+^ and amiloride were added in the presence of external 1.2 mM Ca^2+^. All data are the mean ± SEM of percentage change compared to the control condition in normal Ca^2+^. Asterisks indicate statistical significance: ***p < 0.001 vs. control, as evaluated by one-way ANOVA.

**FIGURE 3:**
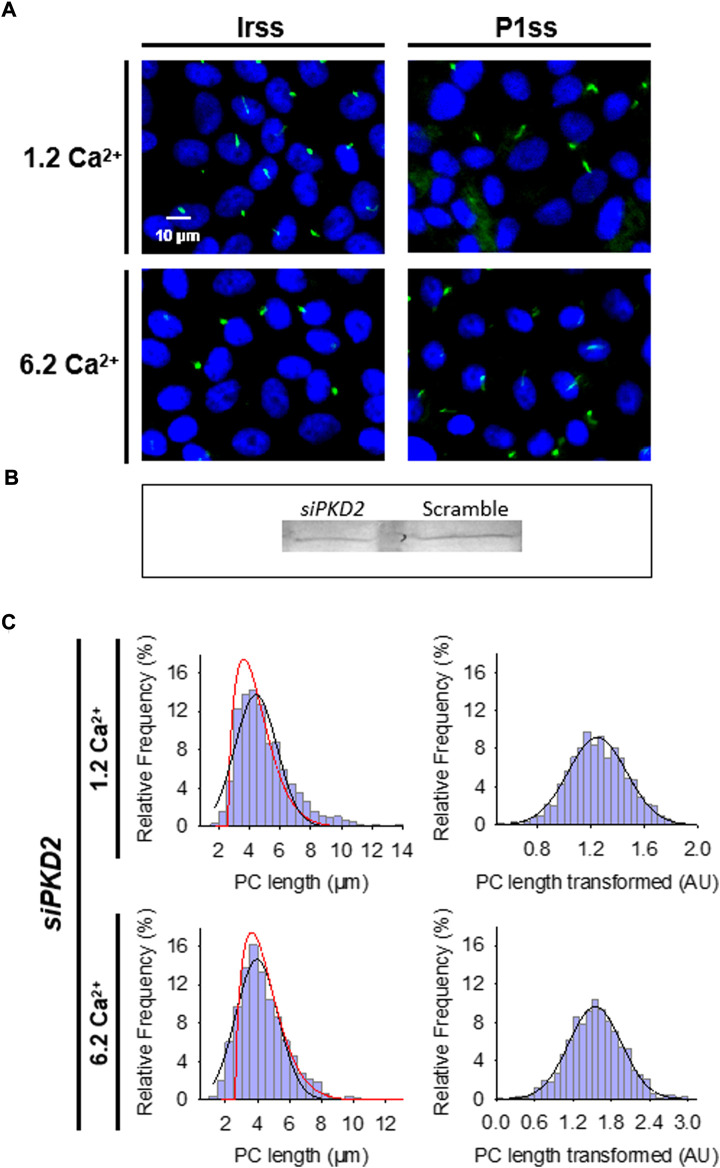
Effect of PKD2 gene silencing on the primary cilium length of wild-type LLC-PK1 cells. **(A)** Primary cilia are observed in green (FITC) and nuclei stained in blue (DAPI) in cells transfected with scrambled (Irss) and PKD2-specific (P1ss) probes in either normal (1.2 mM) or high Ca^2+^ (6.2 mM) conditions. In normal Ca^2+^, P1ss silenced cells had longer primary cilia than their respective controls (Irss, 1.2 Ca). Longer primary cilia were also observed in silenced P1ss cells in high Ca^2+^ respect to Irss, 6.2 Ca condition. **(B)** Western blotting conducted with scrambled and specific PC2 siRNA. **(C)** Box-Cox transformation for data of the primary cilium length in LLC-PK1 cells after inhibition of *PKD2* gene expression under 1.2 mM (Top) and 6.2 mM (Bottom) external Ca^2+^ conditions. Frequency data distributions are shown before (Left) and after (Right) Box-Cox transformation. Red lines indicate control distribution.

Experimental data with *n* > 140 measurements/experiment under each condition, were collected from three to four repeats (*N* experiments). Under all tested experimental conditions, primary cilia length measurements followed a non-Normal distribution (Figures 1–3), showing a large dispersion among experiments (e.g., range of 3.40–11.2 μm length for control condition with 1.2 mM Ca^2+^). Thus, data were first analyzed by a non-parametric one-way ANOVA test performed by Kruskal–Wallis ranges to assess differences within and among different conditions. Individual experiments showed similar general tendencies among experimental conditions, although some did not support statistical significance. In such cases, the Mann-Whitney U test further tested individual observed differences. To improve and enhance the statistical analysis, experimental data were also processed by Box-Cox transformation (Box and Cox, 1964) to aid in later post-hoc appropriate parametric statistical tests that required Normality and allowed further testing with the means and SEs of the data. The Box-Cox transformation is defined as a continuous function that varies to the lambda (λ) power (Kutner et al., 2004). Version 8.0 STATISTICA was used to implement these transformations and normalize the variable “PC length” (Transformed Length, AU). The program searched for the appropriate λ by maximum likelihood, such that the error function was minimal. The Box-Cox transformation function used in the present study was:

$$V=\frac{v_{i}^{\lambda }-1}{\lambda }$$ (2)

Where *V* is the result variable of the transformation, *v_i_* represents the variable to be transformed, and *λ* is the transformation parameter. The transformed histograms showed a remarkable change in their symmetry, approaching a Gaussian distribution (Figures 1–3). The Normality of the data was also contrasted by comparing the empirical distribution with Normal distribution graphs (Figures 1–3). Following data transformation, corrected mean ± SEM values were obtained for each experimental condition. Transformed data were compared by one-way parametric ANOVA and subsequent application of the Tukey post-hoc test. Statistical significance of the averaged data was accepted at *p* < 0.05. The Supplementary Material summarizes the statistical analyses that rendered the final comparison of mean ± SEM for the conditions control, normal Ca^2+^ (1.2 mM), and high Ca^2+^ (6.2 mM) conditions.

**FIGURE 4:**
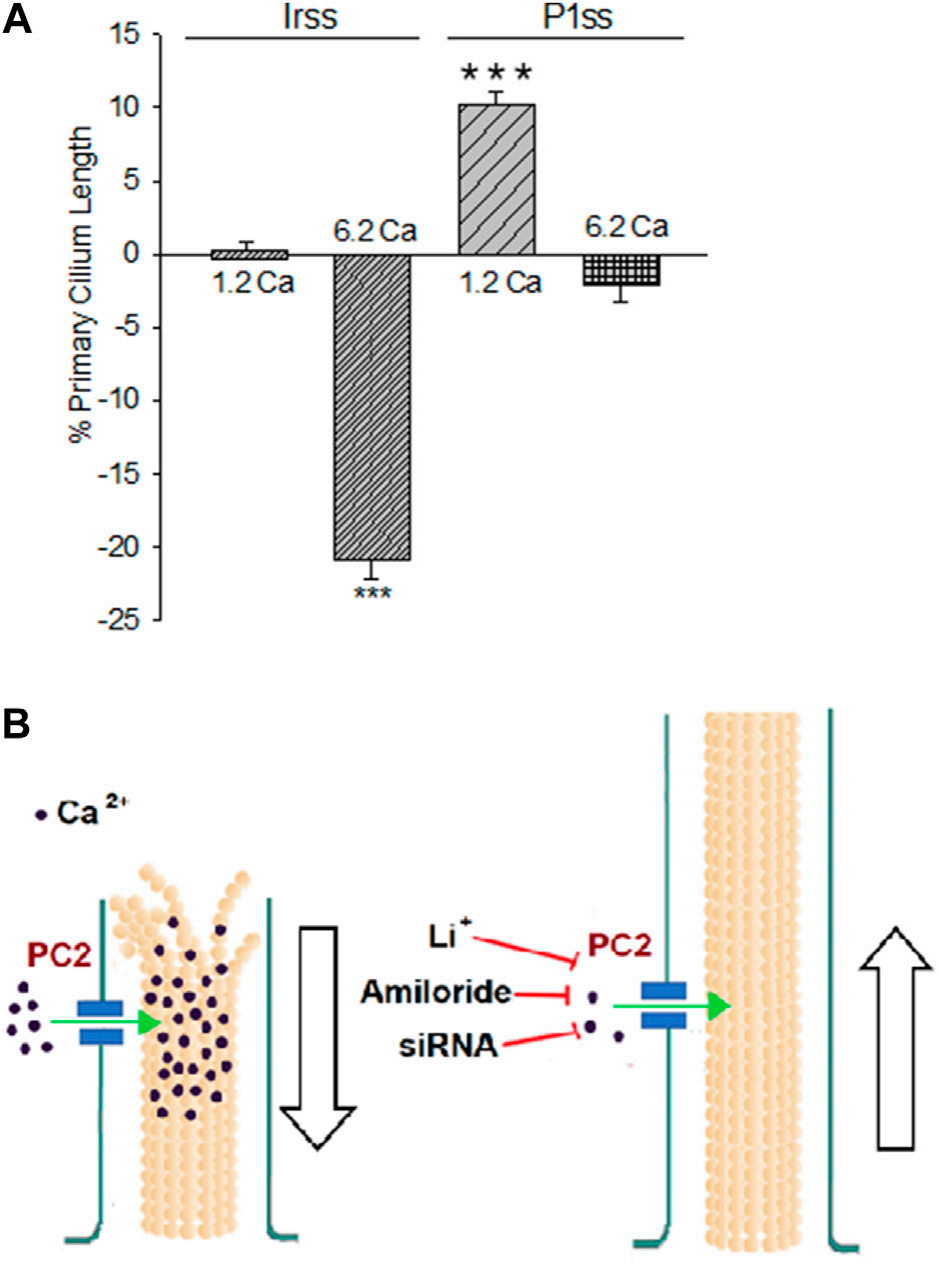
Differences in the primary cilium length after inhibition of PKD2 gene expression. **(A)** Bar graphs represent either the percent increase (positive bars) or decrease (negative bars) of the length of the primary cilium of LLC-PK1 cells in normal (1.2 mM) and high (6.2 mM) external Ca^2+^ concentrations. Data were made relative to the Irss 1.2 mM Ca^2+^, control condition with the scrambled silencing RNA probe (Irss). Irss-treated cells responded to high external Ca^2+^ with a significant decrease in the primary cilium length. Ciliary length of *PKD2*-silenced cells increased to the respective controls in the presence of both normal (1.2 mM) and high (6.2 mM) external Ca^2+^. Asterisks (***) indicate statistical significance at *p* < 0.001 vs. Irss control, evaluated by one-way ANOVA. **(B)** Cartoon of the possible role(s) PC2 plays in regulating the primary cilium length in LLC-PK1 renal epithelial cells. Under normal external Ca^2+^ conditions (1.2 mM), the primary cilium length is maintained by PC2-mediated Ca^2+^ entry. In a high external Ca^2+^ (6.2 mM) concentration, the increased intraciliary Ca^2+^ would help depolymerize axonemal microtubules, reducing their length and thus the primary cilium. Either PC2 inhibition (by Li^+^ or amiloride) or reduction of its expression (siRNA) would instead contribute to reducing Ca^2+^ entry to the primary cilium, also decreasing the rate of MT depolymerization, rendering longer primary cilia.

## 3 Results

### 3.1 Effect of external Ca^2+^ on primary cilia length

Confluent monolayers of wild-type LLC-PK1 cells were exposed overnight to solutions containing either 1.2 mM (normal) or 6.2 mM (high) Ca^2+^ to evaluate the effect of external Ca^2+^ on the length of primary cilia (Figures 1B,C). Cells were fixed and stained (Figure 1A), and Supplemental Material primary cilia length was measured as indicated in Materials and methods (Supplementary Figure S1A). Data did not follow a Normal distribution for any conditions tested (Figure 1C, Left), as expected. Please note that the cells were not cell cycle synchronized for the study. Values were then normalized using the Box-Cox transformation and compared among groups (Figure 1C, Right). See the Supplemental Material for details of the statistical analysis. Cells exposed to high (6.2 mM) external Ca^2+^ had a 13.53 ± 1.25% reduction in primary cilia length as compared to the control condition in normal Ca^2+^ (4.08 ± 0.06 μm, *n* = 653, vs. 4.72 ± 0.05 μm, *n* = 510, *p* < 0.001, respectively) (Figures 1B,C). Thus, changes in external Ca^2+^ regulated the length of primary cilia in LLC-PK1 cells. Although overnight exposure of cells to a Ca^2+^-free medium also decreased primary cilia length (4.25 ± 0.09 μm, *n* = 80), the incubation procedure also produced a dramatic change in cell morphology, suggesting that the phenomenon could occur by mechanisms other than those observed with high Ca^2+^.

### 3.2 Effect of amiloride on the length of primary cilia

The PC2 channel blocker (González-Perrett et al., 2001), amiloride, was tested on the LLC-PK1 cells to explore whether PC2 function may be implicated in regulating primary cilia length. Confluent wild-type LLC-PK1 cells were exposed overnight to a serum-free medium containing normal Ca^2+^ and 200 μM amiloride (Figure 2A). Cells exposed to amiloride had a consistent and statistically significant 33.24 ± 1.15% increase in primary cilia length (6.29 ± 0.05 μm, *n* = 509 vs. 4.72 ± 0.05 μm, n = 510, *p* < 0.001, Figures 2B,C).

### 3.3 Effect of Li^+^ on primary cilia length

To further explore the effect of PC2 inhibition on the primary cilia length, cells were also exposed to Li^+^ (10 mM), which increases ciliary length in neurons and other cells (Miyoshi et al., 2009; Miyoshi et al., 2011), and has a strong inhibitory effect on PC2 channel function (Cantero and Cantiello, 2011). The incubation procedure was similar to that used for amiloride, where the medium, in this case, was instead supplemented with 10 mM LiCl (Figure 2A). Exposure of cells to Li^+^ also induced a statistically significant 17.43% ± 1.48% increase in primary cilia length (5.55 ± 0.07 μm, *n* = 240 vs. 4.72 ± 0.05 μm, *n* = 510, *p* < 0.001, Figures 2B,C).

### 3.4 Effect of inhibition of PC2 expression on primary cilia length

To further confirm the effect of PC2 function on primary cilia length, its expression was reduced by specific PC2 siRNA transfection, as recently reported (Dai et al., 2017). Transfection efficacy was evaluated using a fluorescent silencing probe. It is important to note that scrambled silencing probe transfection (Irss) itself had an effect on primary cilium length (4.23 ± 0.04 μm, *n* = 975 vs. 4.72 ± 0.05 μm, *n* = 510, *p* < 0.001), such that comparisons of PC2-silenced cells (P1ss) were always made with respect to the Irss control condition and the not with untreated cells. In normal Ca^2+^ (Figures 3A,C), P1ss cells had statistically significant 10.25 ± 0.88% longer primary cilia as compared to cells transfected with scrambled RNA (Irss) (4.66 ± 0.04 μm, *n* = 1116 vs. 4.23 ± 0.04 μm, n = 957, *p* < 0.001, Figure 3C, Figure 4A). Interestingly, in the presence of high external Ca^2+^, P1ss cells showed a 24.67 ± 1.65% increase in primary cilia length as compared to the Irss-treated cells exposed to the same external Ca^2+^ concentration (4.14 ± 0.05 μm, *n* = 924 vs. 3.35 ± 0.04 μm, *n* = 813, *p* < 0.001, Figures 3C, Figure 4A). Altogether, the results suggest that inhibition of PC2 expression produces a primary cilium elongation in normal Ca^2+^. This phenomenon is consistent with the effect of both PC2 channel blockers. However, the effect of PC2 expression was Ca^2+^ dependent. In high Ca^2+^ the *PKD2* silenced cells reversed the observed decrease in primary cilia length. Primary cilia length correlated with both the presence of a functional PC2 and the external Ca^2+^ concentration.

## 4 Discussion

The present study provides evidence that a reduction of PC2 function (amiloride and lithium) or expression (silencing) regulate the length of the primary cilia in renal epithelial cells, in a Ca^2+^ dependent manner (Figure 2C). It is known that Ca^2+^ is a potent microtubular (MT) depolymerizing agent (Karr et al., 1980; O'Brien et al., 1997). Thus, the simplest possible working hypothesis is that PC2, a Ca^2+^-permeable ion channel, might help control the delivery and concentration of ciliary Ca^2+^. High external Ca^2+^ resulted in the shortening of primary cilia, which is consistent with the catastrophic depolymerization of axonemal MTs enabled by the PC2-mediated increase in intraciliary Ca^2+^. However, it is important to note that cells incubated in the absence of external Ca^2+^ also displayed shorter primary cilia (10% reduction). While there is no apparent explanation for this phenomenon, it is possible that cells exposed for several hours to a Ca^2+^-free environment would suffer several changes, including major rearrangements in the cytoskeleton, and loss of adhesiveness to the substrate, leading to the retraction of the primary cilia. Under normal external Ca^2+^ conditions (1.2 mM), the primary cilium length is maintained by PC2-mediated Ca^2+^ entry. In a high external Ca^2+^ (6.2 mM) concentration, the increased intraciliary Ca^2+^ reduced the length of the primary cilium. PC2 inhibition by treatment with either Li^+^ or amiloride or a reduction of its expression (siRNA) would instead contribute to reducing Ca^2+^ entry to the primary cilium, also decreasing the rate of MT depolymerization, rendering longer primary cilia. The present study suggests that PC2 mediated Ca^2+^ transport is a key mechanism to the control of primary cilium length in renal epithelial cells.

Despite the correlation between PC2 expression/function and cilia length observed in this study and the unequivocal primary cilium implications in ADPKD cystogenesis, there is practically no information available on the role cilia length plays in human *PKD* tissues. The disease is largely associated with mutations in the *PKD1* gene, as compared to *PKD2*, such that essential aspects to the regulation of the PC2 channel may be implicated and not observed in normal tissue. A recent study by Zhou’s group (Shao et al., 2020) showed that Ift88 inactivation suppressed cystogenesis. The study also showed cilia elongation in human and mouse ADPKD kidneys and that cilia loss was not necessary for suppressing cystogenesis in *PKD*. This is perhaps the only attempt at addressing the connection between primary cilium length in the human disease and the association between polycystins and primary cilia. The study further linked primary cilia length with cyst size in human ADPKD patients, and disease progression and signaling in ADPKD mouse models. The authors showed that primary cilia shortening was sufficient to suppress cystogenesis. *Pkd2ws25*/− knockout mice displayed marginally shorter primary cilia in the kidneys, and longer primary cilia in *Pkd2flox/flox* knockout mouse overexpressing Arl13b. The authors concluded that changes in primary cilium length could not be solely attributed to *Pkd1* or *Pkd2* mutations. The study observed that the primary cilium is elongated in kidneys from patients with ADPKD and that this elongation coincides with disease progression (Shao et al., 2020). Shortening cilia in two orthologous models of ADPKD by genetic removal of intraflagellar transport 88 strikingly retarded the cystic disease in the kidney and liver (Shao et al., 2020). Recent evidence in lower organisms, including *Tetrahymena* and *Chlamydomonas* (Perrone et al., 2003; Cao et al., 2009; Waclawek et al., 2017; Jiang et al., 2019; Li et al., 2020; Maurya and Sengupta 2021; Park et al., 2021), and sensory neurons of *Caenorhabditis elegans* (Evans et al., 2006; Hao et al., 2011; Morsci and Barr 2011; van der Vaart et al., 2015; Prevo et al., 2017; Shanmugam et al., 2018; Maurya et al., 2019), implicate the intraflagellar transport, and most prominently phosphorylating pathways in the regulation of ciliary length, which are likely independent of the PC2 channel complex but associated to the control of microtubule assembly (Waclawek et al., 2017; Maurya et al., 2019; Li et al., 2020).

In the present study we modulated ciliary length with drugs of clinical importance (Miyoshi et al., 2011). We tested the effect of PC2 inhibition on ciliary length with amiloride (or N-amidino-3,5-diamino-6-chloropyrazinecarboxamide) (Kleyman and Cragoe, 1988), a diuretic that increases renal excretion of Na^+^ while decreasing K^+^ excretion (Palmer and Andersen, 1989). Amiloride and its derivative Benzamil are high-affinity blockers of the epithelial Na^+^ channel, ENaC, acting as a blocking agent of its pore (Palmer and Andersen, 1989). Amiloride also functions as an inhibitor of several Na^+^ transporters and nonselective cation channels, including Na^+^/Ca^2+^, and Na^+^/H^+^ exchangers, nonselective cation channels, and voltage-gated K^+^ and Ca^2+^ channels (Kleyman and Cragoe, 1988; Kleyman and Cragoe, 1990; Garcia et al., 1990; Sariban-Sohraby and Benos, 1986; Tytgat et al., 1990; Doi and Marunaka, 1995; Murata et al., 1995; Stoner and Viggiano, 2000; Hirsh, 2002; Castañeda et al., 2019), including PC2 (González-Perrett et al., 2001), and PC2-like (TRPP3) channel (Dai et al., 2007). The treatment with amiloride at a concentration expected to produce a complete inhibition of the PC2 channel (200 μM, González-Perrett et al., 2001) produced an increase in ciliary length, although it is possible for amiloride to block other Ca^2+^ entry mechanisms or channels such as ciliary ENaC (Raychowdhury et al., 2005) that may affect ciliary length independently. The effect of amiloride is in agreement with the effect of LiCl that inhibits PC2 function (Cantero and Cantiello, 2011) and also produced statistically longer primary cilia, agreeing with other studies where Li^+^ treatment increased ciliary length in various cell models (Miyoshi et al., 2009; Miyoshi et al., 2011).

Treatment with Li^+^ is a critical pharmacotherapy as a proven agent in bipolar disorder and mania and, more recently, in psychoses and various neurodegenerative diseases (Machado-Vieira et al., 2009). Although it is not yet clear how Li^+^ ion exerts its biological effect(s), a lengthening of the primary cilium has been observed in various cultured cells, including neurons and fibroblasts (Miyoshi et al., 2009; Miyoshi et al., 2011). The primary cilium is a preferential location for PC2 expression (Nauli et al., 2003; Raychowdhury et al., 2005), and we have shown that Li^+^ reduces PC2 channel conductance and modifies the reversal potential of the *in vitro* translated PC2 (Cantero and Cantiello, 2011). Even low concentrations of Li^+^ (1–10 mM) have a considerable effect on PC2 function (Cantero and Cantiello, 2011), suggesting the potential relevance of this ion in a clinical setting. At least two mechanistic aspects should be considered in this regard. Li^+^ could be transported to the ciliary compartment, accumulating and exerting a still unknown structural effect on the ciliary length even at low concentrations. Another possibility is to consider the potential impact of the Li^+^ interaction with PC2 on Ca^2+^ transport. In any case, Li^+^ blockade of PC2 in organelles such as the primary cilium may help explain the impaired sensory function and the therapeutic effects of Li^+^.

*PKD2* gene expression was silenced in the LLC-PK1 cells to prove the functional role of PC2 in regulating the length of the primary cilium. In the presence of normal Ca^2+^, *PKD2*-silenced cells showed a longer primary cilium than their respective controls. This is consistent with the fact that the absence of PC2 would have a similar effect to that of PC2 inhibitors, giving rise to longer primary cilia and suggesting that PC2 is necessary for the regulatory Ca^2+^ entry into the organelle. Interestingly, in the presence of high external Ca^2+^, PC2-silencing induced a reversal such that the values were similar to the control cells incubated in normal Ca^2+^. Although there is no explanation for this phenomenon, it may be related to PC2 silencing, which only represented approximately 50% of the inhibited gene product. It is possible that the remaining gene product could compensate and maintain a sustainable Ca^2+^ influx to the primary cilium. In the absence of PC2 Ca^2+^ transport could also be potentiated by other Ca^2+^-permeable channels in the primary cilium, including TRPC1 and TRPV4 (Raychowdhury et al., 2005; Ma et al., 2010). PC2 interacts with these TRP channel isotypes from different hetero-multimeric complexes. PC2/TRPC1 hetero-multimeric channels, for example, show different conductance patterns than their homomeric counterparts (Zhang et al., 2009) and may modify their Ca^2+^ permeability properties, although information in this regard is yet unavailable. PC2 silencing could affect these interactions, thus interfering with the mechano-transduction of the Ca^2+^ signals associated with primary cilia.

Ca^2+^ and cAMP signaling regulate PC2 channel activity (Cantero and Cantiello, 2013; Cantero et al., 2015). However, little is known as to how Ca^2+^ and cAMP signals interact with each other leading to changes in the primary cilium length. We also observed how cAMP signals regulate the length of the primary cilium in confluent wild-type LLC-PK1 renal epithelial cells (Scarinci et al., 2020). Treatment of the cells with the cAMP analog 8-Br-cAMP in normal (1.2 mM) and high (6.2 mM) external Ca^2+^ produced an increase in the length of the primary cilium. This is apparently paradoxical because cAMP signaling and PKA activation both stimulate PC2 channel function. Thus, PC2 may not be the only Ca^2+^-controlling pathway. Other transport mechanisms may also participate in the regulation of the Ca^2+^ concentration in the primary cilium, controlling its length. Interestingly, however, exposure of the cells to vasopressin, which increases cAMP in the primary cilia of LLC-PK1 cells, mimicked the effect of 8-Br-cAMP in normal but not in high Ca^2+^. Thus, the study determined a complicated crosstalk between the cAMP and Ca^2+^ signals to modulate the length of the primary cilium, in a phenomenon that implicates the presence of PC2. Besschetnova et al. (2010) showed that increases in Ca^2+^ and cAMP increase the length of embryonic kidney cells. Although the primary cilium could be considered a compartment largely independent of the rest of the cell, both second messenger signals affect the entire cytoplasm and not solely the primary cilium. Expected changes in the concentrations of Ca^2+^ and cAMP may be associated with the activation of cytoplasmic reactions including Ca^2+^-dependent phosphodiesterases and adenylyl cyclases that also regulate ciliary length.

## 5 Conclusion

In conclusion, our results indicate that a functional PC2 controls the length of the primary cilium in renal epithelial cells, such that pathways that lead to its inhibition, including gene suppression and possibly a consequent decrease in ciliary Ca^2+^ entry, render ciliary elongation. In contrast, high external Ca^2+^ leads to a reduction in its length (Figure 4B). These results open the possibility of elucidating the relationship between changes in ciliary length and Ca^2+^ influx in the onset of ciliopathies and the formation of signals leading to renal cysts, more specifically, in ADPKD.

## Data Availability

The raw data supporting the conclusions of this article will be made available by the authors, on a reasonable request.
