# Supplementary material for: Polycystin-2 (TRPP2) regulates primary cilium length in LLC-PK1 renal epithelial cells
Source: Front Physiol. 2022 Oct 4;13:995473. doi: 10.3389/fphys.2022.995473 (PMC9577394; doi:10.3389/fphys.2022.995473)
Supplement: Supplementary file 1 [file DataSheet1.docx]

Supplementary Material

# Supplementary Figures and Tables

The statistical analyses of the experimental data were as follows. Data from -either three or- four experiments were collected for any given condition (in the present example two conditions), 1.2 mM and 6.2 mM external Ca^2+^ concentrations. Data were shown to be wide variable for the ciliary length measurements, even when cells were grown and kept undertissue culture conditions until total confluence. This variability made difficult any comparisons among groups. The incubation in high Ca^2+^ produced a decrease in ciliary length as compared to cells kept in normal Ca^2+^ (1.2 mM). This trend was observed for all four repeats (Exps 1-4, See Table S1, and Fig. 1A). Because no data followed a Normal distribution, the non-parametric one-way ANOVA test was first performed by Kruskall-Wallis ranges, for the 1.2 and 6.2 mM Ca^2+^ conditions. The results indicated that in normal Ca^2+^, experiments 3 and 4 were not significantly different, but the others were (p < 0.001). In high Ca^2+^, in contrast, no significant differences were found between experiments 1 and 2 and between experiments 3 and 4. Table S1 shows the median and the 25% and 75% quartiles obtained under both conditions for each one of the experiments.

**Supplementary Table 1. Ciliary length values for cells exposed to different concentrations of external Ca^2+^. Data are the medians and the 25% and 75% quartiles.**

|  | **Median, Q1 and Q3, µm** | | | |
| --- | --- | --- | --- | --- |
| **[Ca^2+^], mM** | **Exp 1** | **Exp 2** | **Exp 3** | **Exp 4** |
| 1.2 | **9.69** (7.52, 11.2) (*n* = 140) | **6.27** (4.69, 7.77) (*n* = 159) | **4.08** (3.40, 5.82) (*n* = 153) | **4.18** (3.60, 4.85) (*n* = 158) |
| 6.2 | **7.15** (4.68, 9.29) (*n* = 129) | **5.81** (5.07, 7.01) (*n* = 202) | **3.09** (2.62, 3.57) (*n* = 172) | **3.10** (2.75, 3.68) (*n* = 150) |

*The medians, Q1 and Q3, are observed for ciliary length at two different Ca^2+^ concentrations (1.2 and 6.2 mM Ca^2+^), with their respective number of cases “n”.*

To assess statistical differences between experimental conditions (in this example the two Ca^2+^ concentrations) for each particular experiment, the Mann-Whitney U test was used instead. The results showed significant differences between experiments 1, 3 and 4 (p < 0.001), but not for experiment 2 (data not shown). Thus, we sought to normalize the data groups under each experimental condition, in order to apply parametric tests.

To be able to compare means and Standard Errors between groups and the subsequent statistical analysis with more powerful tests, data were normalized by Box-Cox transformation (see main text, Figs. 1-4). From the transformation formula (see Methods), the value of the mean with its SEM was obtained for each incubation condition. The one-way ANOVA parametric test was then performed. Table S2 shows the mean ± SEM for normalized values under both Ca^2+^ concentrations. For the 1.2 Ca^2+^ condition, only experiments 3 and 4 showed no significant differences between each other, as with the Kruskall-Wallis test. In the 6.2 mM Ca^2+^ condition, experiments 3 and 4 showed no significant differences, but differences were observed for all others (p < 0.001).

**Supplementary Table 2. Ciliary length values for cells exposed to different concentrations of external Ca^2+^. Data are the mean ± SEM after Box-Cox re-transformation of original data**

|  | **Mean** **± SEM** | | | |
| --- | --- | --- | --- | --- |
| **[Ca^2+^], mM** | **Exp 1** | **Exp 2** | **Exp 3** | **Exp 4** |
| 1.2 | 9.66 ± 0.22 (*n* = 140) | 6.12 ± 0.11 (*n* = 159) | 4.08 ± 0.10 (*n* = 153) | 4.18 ± 0.10 (*n* = 158) |
| 6.2 | 6.96 ± 0.25 (*n* = 129) | 5.83 ± 0.08 (*n* = 202) | 3.03 ± 0.08 (*n* = 172) | 3.12 ± 0.09 (*n* = 150) |

*Means ± SE were observed at different concentrations of Ca^2+^ (1.2 and 6.2 mM Ca^2+^) with their respective n.*

Once the data were re-transformed, the tendency to reduce ciliary length after incubation in 6.2 Ca again was observed. This was evident in the four experiments (Fig S1). The Student *t* test statistic showed significant differences for each of the comparisons between the 1.2 Ca and 6.2 Ca conditions (p < 0.03 for experiment 2 and p < 0.001 for experiments 1, 3 and 4).

## Supplementary Figures

The length of extended primary cilia on the fixed confluent monolayer was manually measured from 2D images with the image analysis program ImageJ (NIH software), as described elsewhere (Ou et al., 2009; Besschetnova et al., 2010; Miyoshi et al. 2009; Sipos et al., 2018). Briefly, FITC-labeled fluorescent primary cilia were traced with the “Freehand Line” tool of the software to obtain its length in pixels. The results were then converted into mm by calibration with a Neubauer chamber (Hausser Scientific, Horsham, PA, USA) (Fig. 1A). Although this technique risks a selection bias, it is considered reliable, allowing the measurement of many irregular primary cilia and facilitating their subsequent statistical analyses.


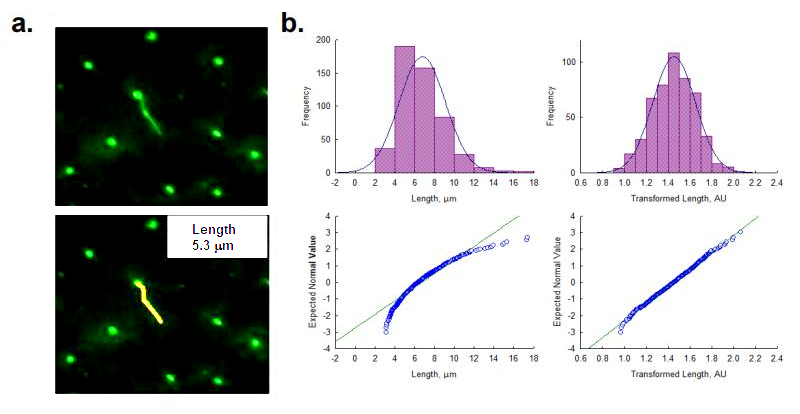


**Fig. 1A:** *Primary cilia inmunolabeling and measurement.* **a.** Primary cilia were identified (x40) and measured with the IPLab software. **b.** Top. Data distribution (Frequency) histograms before (Left panel) and after the Box-Cox transformation for the variable “Length of the primary cilium” are shown before and after Box-Cox transformation. The symmetry reached after the transformation is consistent with a Normalized distribution. *Bottom*. Probability density function plot before (Left) and after Box-Cox transformation. Transformed data points (Blue) are closer to the fitted Normal distribution line (Green).


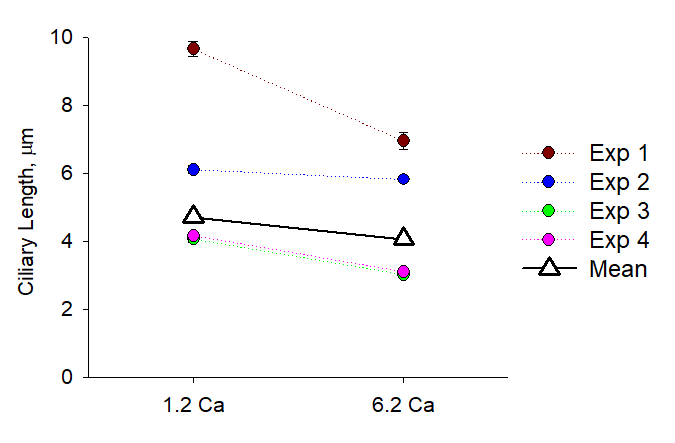


**Figure 2A.** Effect of external Ca^2+^ on ciliary length for each one of the four experiments after Box-Cox re-transformation of original data. Statistical difference of individual experiments evaluated by Student t test was as follows, exps, 1, 3, and 4 p < 0.001, and exp 2, p < 0.05, with respect to the control condition. Please note that open triangles represent the average for the four experiments for both conditions. Statistical significance is indicated in the text.

Fig. 2A shows (open triangles) pooled average of normalized data for the four experiments under each condition, rendering the values reported in the manuscript, 4.72 ± 0.05 μm, n = 510 vs. 4.08 ± 0.06 μm, n = 653, p < 0.001, for the 1.2 and 6.2 mM Ca^2+^, respectively. In the same way that incubating the cells in 6.2 mM Ca^2+^ produced a reduction of primary cilia length, which is reported by the pooled data, a similar approach was conducted for allremaining conditions. Namely, after evaluating each of the experiments among themselves, values were included for each condition in a common pool after Box-Cox transformation, using mean ± SEM for the subsequent application of parametric statistical tests. All experimental groups showed strong Normalization and a significant statistical difference as compared to its own respective control condition (see main manuscript).

**
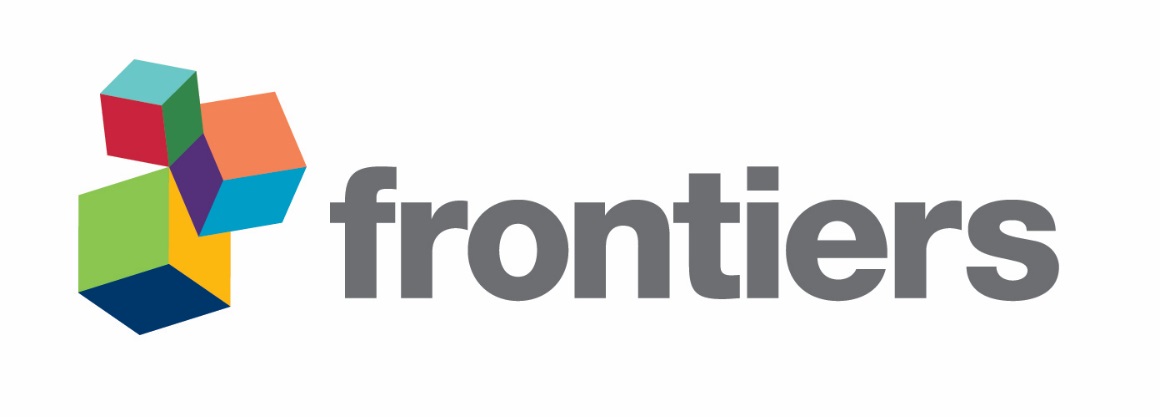
**
